# Appraisal of International Guidelines for Cutaneous Melanoma Management using the AGREE II assessment tool

**DOI:** 10.1016/j.jpra.2021.11.002

**Published:** 2021-12-08

**Authors:** C. Jacklin, M. Tan, S. Sravanam, C.J. Harrison

**Affiliations:** 1 Medical Sciences Divisional Office, University of Oxford, Level 3, John Radcliffe Hospital, Oxford OX3 9DU, UK; 2 Academic Section of Vascular Surgery, Department of Surgery and Cancer, Imperial College, London; 3 Nuffield Department of Orthopaedics, Rheumatology and Musculoskeletal Sciences, University of Oxford, Oxford, UK

**Keywords:** Melanoma, Practice Guideline, Margins of Excision, Radiotherapy, Chemotherapy

## Abstract

**Background:**

The evidence base behind new melanoma treatments is rapidly accumulating. This is not necessarily reflected in current guidance. A recent UK-based expert consensus statement, published in JPRAS, has called for updates to the widely accepted 2015 National Institute for Health and Care Excellence (NICE) guideline for melanoma (NG14). We aimed to compare the quality of NG14 to all other melanoma guidelines published since.

**Methods:**

We conducted a systematic search of PubMed, Medline, and online clinical practice guideline databases to identify melanoma guidelines published between 29th July 2015 and 23rd August 2021 providing recommendations for adjuvant treatment, radiotherapy, surgical management, or follow-up care. Three authors independently assessed the quality of identified guidelines using the Appraisal of Guidelines for Research & Evaluation Instrument II (AGREE II) assessment tool, which measures six domains of guideline development. Inter-rater reliability was assessed by Kendall's coefficient of concordance (W).

**Results:**

Twenty-nine guidelines were included and appraised with excellent concordance (Kendall's W for overall guideline score 0.88, p<0.001). Overall, melanoma guidelines scored highly in the domains of ‘Scope and purpose’ and ‘Clarity of presentation’, but poorly in the ‘Applicability’ domain. The NICE guideline on melanoma (NG14) achieved the best overall scores.

**Conclusion:**

Melanoma treatment has advanced since NG14 was published, however, the NICE melanoma guideline is of higher quality than more recent alternatives. The planned update of NG14 in 2022 is in demand.

## Introduction

Melanoma treatment options are rapidly evolving^1^. Checkpoint and **v-raf murine sarcoma viral oncogene homolog B1 (**BRAF) inhibitors have significantly improved survival rates in advanced disease^2^, ^3^, ^4^, ^5^, ^6^, ^7^, and recent high profile trials have challenged previous approaches to lymph node and skin surgery^8^, ^9^, ^10^, ^11^, ^12^, ^13^. In a rapidly advancing field, guidelines quickly become outdated. The National Institute for Health and Care Excellence (NICE) is internationally renowned for its rigorous, multi-stakeholder approach to guideline development. However, a recent consensus statement of UK melanoma experts has challenged the widely adopted 2015 NICE guidance for melanoma (NG14)^14^ in light of landmark trials published over the last five years, including **Multicenter Selective Lymphadenectomy Trial II (MSLT-II)** and the Dermatologic Cooperative Oncology Group-Selective Lymphadenectomy Trial (**DeCOG**-SLT)^8^^,^^9^^,^^15^.

The quality of guidelines can be assessed according to the Appraisal of Guidelines for Research and Evaluation II (AGREE II) assessment tool, a widely accepted instrument for guideline quality appraisal, with established construct validity^16^, ^17^, ^18^. The AGREE II assessment tool evaluates the quality and reporting of practice guidelines using 23 items across six domains, namly ‘Scope and purpose’, ‘Stakeholder involvement’, ‘Rigour of development’, ‘Clarity of presentation’, ‘Applicability’, and ‘Editorial independence’. Each item is scored on an ordinal scale from 1 (strongly disagree) to 7 (strongly agree) according to AGREE II manual^16^ and an additional overall score is assigned to each guideline.

The objective of this study was to systematically appraise the quality of melanoma guidelines developed since the NG14 was published, and compare these more recent alternatives to NG14, using the AGREE II criteria.

## Methods

### Protocol and registration

The study protocol was pre-registered on the Open Science Framework^19^ and conducted in line with the Preferred Reporting Items for Systematic Reviews and Meta-Analyses (PRISMA) statement^20^.

### Search strategy

The search strategy was designed with the assistance of a search strategist (Suppl. 1). PubMed and Medline databases were searched from 29th July 2015 until 23rd August 2021.

**Figure 1:**
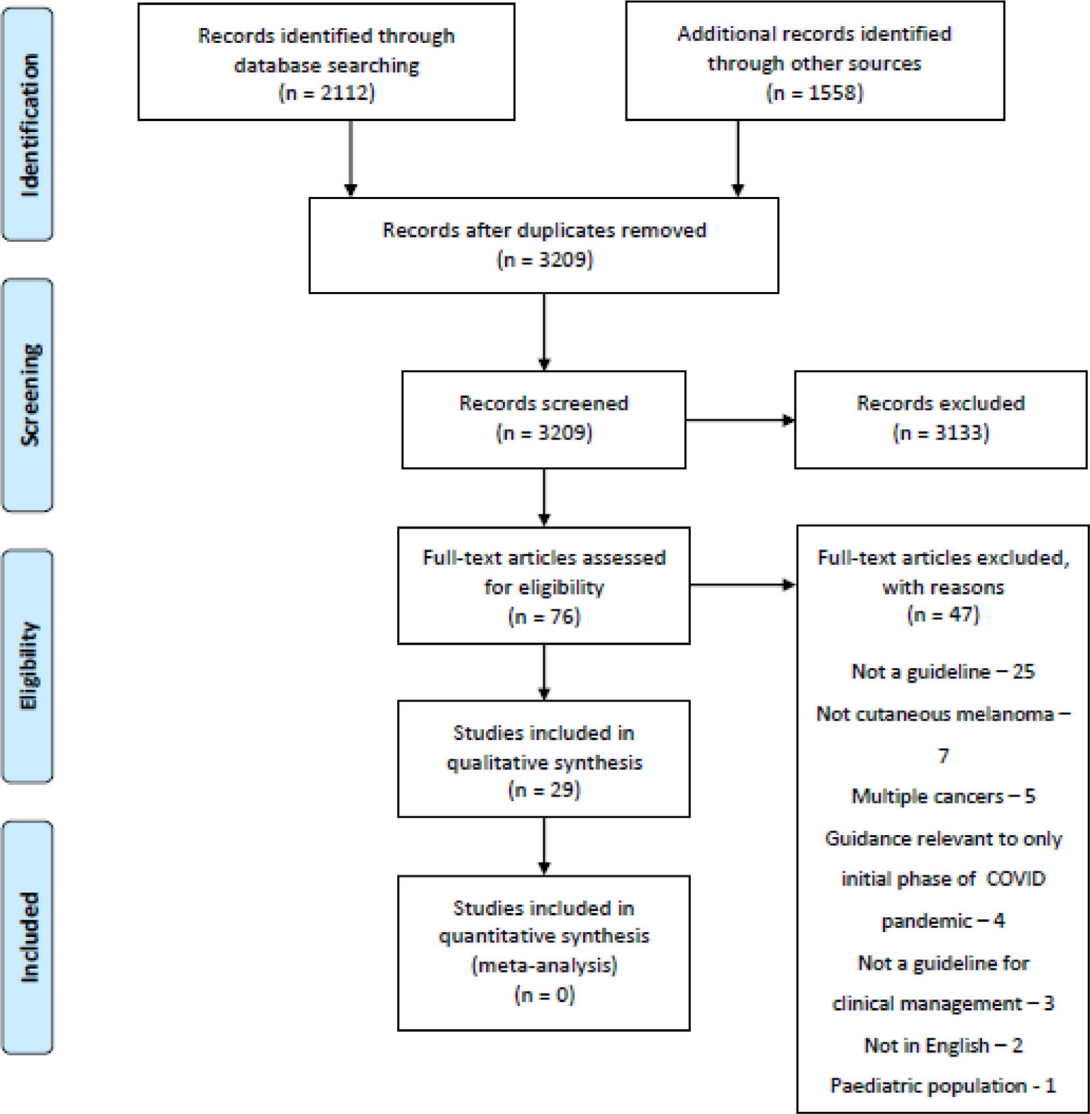
Preferred Reporting Items for Systematic Reviews and Meta-Analyses (PRISMA) flow diagram.

Additionally, the following clinical practice guidelines databases were searched with the search keywords: “melanoma”: National Institute for Health and Care Excellence; Canadian clinical practice guidelines InfoBase: Clinical Practice Guidelines Database; Scottish Intercollegiate Guidelines Network; Australian Clinical Practice Guidelines; and Guidelines International Network. A further search was carried out in the Turning Research into Practice (TRIP) database with the search term “melanoma” followed by using the filter tools: “guidelines” and “since 2015”. Search results were screened by an author CJ (Fig. 1).

### Eligibility criteria

Results from the search were included if they provided recommendations on at least one of the following: adjuvant treatment, radiotherapy, surgical management, or follow-up care for cutaneous melanoma, and were developed after the publication of the NG14 (29th July 2015).

Publications were excluded if they were not in the English language, were for the pediatric population only, were aimed at nurses only, provided guidelines for multiple cancers, and recommendations were relevant only to care during the initial phase of the COVID-19 pandemic.

### AGREE II assessment

Three assessors independently appraised the candidate guidelines for malignant melanoma management using the “My AGREE PLUS” platform^21^. Guidelines were assigned ratings on an ordinal 1-7 scale for 23 items across six domains. Assessors also assigned a global rating out of seven scales and provided an overall judgment on the appropriateness of the guidelines for use with or without modifications.

To aid better interpretation, overall scaled percentage scores were calculated for each item, domain, and guideline, by summing the scores of individual assessors and presenting them as a percentage of the maximum attainable score. To do this, we used the calculation specified in the AGREE II user manual^16^. We calculated inter-observer reliability using both Fleiss kappa and Kendall's coefficient of concordance (W).

## Results

### Guideline Search

A total of 3670 articles were identified by the search strategy, of which 461 duplicates were removed. The remaining 3209 articles were screened by their title and abstract; during screening 3133 articles were excluded. Next, 76 full-text articles were assessed for eligibility, of them again were excluded (justifications are provided in Fig. 1), leaving 29 articles^14^^,^^15^^,^^22^, ^23^, ^24^, ^25^, ^26^, ^27^, ^28^, ^29^, ^30^, ^31^, ^32^, ^33^, ^34^, ^35^, ^36^, ^37^, ^38^, ^39^, ^40^, ^41^, ^42^, ^43^, ^44^, ^45^, ^46^, ^47^, ^48^, ^49^, ^50^ for appraisal with the AGREE II tool. A summary of the characteristics of the articles appraised in this review is presented in Table 1.

**Table 1:** Scaled guideline percentage scores and overall ratings.

Title	Year Published	Author	Scaled guideline percentage score (%)	Overall judgment - fit for purpose?
Melanoma Assessment and Management^14^	2015	National Institute for Health and Care Excellence, UK	94.2	Yes
SIGN 146 - Cutaneous Melanoma^50^	2017	Scottish Intercollegiate Guidelines Network, Scotland	89.4	Yes
Clinical Practice Guidelines for the Diagnosis and Management of Melanoma^48^	2020	Cancer Council, Australia	80.4	Yes
Systemic Therapy for Melanoma: ASCO Guideline^24^	2020	American Society of Clinical Oncology, US	80.4	Yes
Sentinel Lymph Node Biopsy and Management of Regional Lymph Nodes in Melanoma: American Society of Clinical Oncology and Society of Surgical Oncology Clinical Practice Guideline Update^44^	2017	American Society of Clinical Oncology and Society of Surgical Oncology, US	79.7	Yes
Primary Excision Margins and Sentinel Lymph Node Biopsy in Cutaneous Melanoma^38^	2017	Cancer Care Ontario, Canada	78.5	Yes
Follow-up of Patients with Cutaneous Melanoma who were treated with Curative Intent^38^	2015	Cancer Care Ontario, Canada	75.8	Yes
Locoregional management of in-transit metastasis in melanoma^25^	2020	Cancer Care Ontario, Canada	75.8	Yes
Systemic Adjuvant Therapy for Adult Patients at High Risk for Recurrent Cutaneous or Mucosal Melanoma: An Ontario Health (Cancer Care Ontario) Clinical Practice Guideline^49^	2020	Cancer Care Ontario, Canada	75.1	Yes
The Use of Adjuvant Radiation Therapy for Curatively Resected Cutaneous Melanoma^31^	2016	Cancer Care Ontario, Canada	72	Yes
Guidelines of Care for the Management of Primary Cutaneous Melanoma^45^	2018	American Academy of Dermatology, US	66.4	Yes with modifications
An Update on the Society for Immunotherapy of Cancer Consensus Statement on Tumor Immunotherapy for the Treatment of Cutaneous Melanoma: Version 2.0^43^	2018	Society for Immunotherapy of Cancer	65.9	Yes with modifications
Japanese Dermatological Association Guidelines: Outlines of Guidelines for Cutaneous Melanoma 2019^41^	2019	Japanese Dermatological Association, Japan	64.7	Yes
European Consensus-Based Interdisciplinary Guideline for Melanoma. Part 2: Treatment e Update 2019^42^	2019	European Dermatology Forum, European Association of Dermato-Oncology, European Organization for Research and Treatment of Cancer	60.4	Yes
Sentinel Node Biopsy in Primary Cutaneous Melanoma^29^	2016	Alberta Health Services, Canada	56.3	Yes
Guidelines of the Brazilian Dermatology Society for Diagnosis, Treatment and Follow Up of Primary Cutaneous Melanoma – Part I and Part II^36^^,^^37^	2015	Brazilian Dermatological Society, Brazil	55.1	Yes with modifications
French Updated Recommendations in Stage I To III Melanoma Treatment and Management^35^	2017	Guillot et al.	53.4	Yes with modifications
Systemic Anti-Cancer Therapy of Patients with Metastatic Melanoma^28^	2017	National Cancer Control Programme, Ireland	52.9	Yes with modifications
Cutaneous Melanoma: ESMO Clinical Practice Guidelines for Diagnosis, Treatment and Follow Up^40^	2019	European Society for Medical Oncology	50.7	No
Current Role of Sentinel Lymph Node Biopsy in the Management of Cutaneous Melanoma: A UK Consensus Statement^15^	2020	Peach et al.	50	Yes with modifications
Spanish Multidisciplinary Melanoma Group (GEM) Guidelines for the Management of Patients with Advanced Melanoma^33^	2015	Spanish Multidisciplinary Melanoma Group, Spain	42.8	No
Cutaneous Melanoma, Version 2.2019^39^	2019	National Comprehensive Cancer Network, US	42.3	Yes with modifications
ESMO consensus conference recommendations on the management of locoregional [and metastatic] melanoma: under the auspices of the ESMO Guidelines Committee^26^^,^^27^	2020	European Society for Medical Oncology	40.3	Yes with modifications
EANM Practice Guidelines for Lymphoscintigraphy and Sentinel Lymph Node Biopsy in Melanoma^34^	2015	European Association of Nuclear Medicine	37	No
Radiological imaging of melanoma: a review to guide clinical practice in New Zealand^22^	2021	Francis et al.	35.7	No
SEOM clinical guideline for the management of cutaneous melanoma^23^	2021	Sociedad Española de Oncología Médica, Spain	32.9	No
The Updated Swiss Guidelines 2016 for the Treatment and Follow-Up of Cutaneous Melanoma^32^	2016	Dummer et al	32.4	No
SEOM Clinical Guideline for the Management of Malignant Melanoma^30^	2017	Sociedad Española de Oncología Médica, Spain	28.7	No
Chinese Guidelines for Diagnosis and Treatment of Melanoma 2018^46^	2018	National Health Commission of the People's Republic of China, China	18.6	No

Four guidelines^51^, ^52^, ^53^, ^54^ were excluded because they provided recommendations relevant to only the temporary disruption to care caused by the initial phase of the COVID-19 pandemic. Examples of their recommendations include emphasizing the importance of in-person examination^52^^,^^53^, review of requirement and/or timing of routine clinics^51^^,^^53^^,^^54^, opting for the longest approved interval between immunotherapy treatments^53^, and deferring SLB^52^^,^^54^.

### Guideline appraisal

Two guidelines were given a global rating of 7/7 by all assessors: NG14 (the 2015 NICE guideline)^14^, and the Scottish Intercollegiate Guidelines Network (SIGN) “SIGN 146: cutaneous melanoma” guideline^50^. The median scaled guideline percentage score (representing all raters’ assessments of a guideline, across all items) was 58.2%. No guideline received the maximum scaled guideline percentage score. The highest guideline percentage score (94%) was awarded to NG14.

### Inter-rater reliability

Fleiss kappa value, assessing agreement of specific numeric ratings, ranged from -0.11 to 0.23 for item scores. Kendell's coefficient of concordance (W), assessing agreement of rankings, ranged from 0.52−0.88 (Table 2).

**Table 2:** Inter-rater reliability statistics for each item (1-23) in AGREE II, judgment (if fit for purpose), and rating of the overall score.

Item	Fleiss Kappa (p value)	Kendall's W (p value)
**1**	0.12 (.030)	0.68 (<.001)
**2**	0.05 (.374)	0.80 (<.001)
**3**	0.11 (.057)	0.80 (<.001)
**4**	0.02 (.678)	0.74 (<.001)
**5**	0.13 (.022)	0.60 (.006)
**6**	0.18 (<.001)	0.71 (<.001)
**7**	0.27 (<.001)	0.86 (<.001)
**8**	0.13 (.004)	0.78 (<.001)
**9**	-0.06 (.232)	0.63 (.003)
**10**	0.12 (.001)	0.81 (<.001)
**11**	<0.01 (.985)	0.52 (.031)
**12**	0.07 (.156)	0.75 (<.001)
**13**	0.23 (.010)	0.82 (<.001)
**14**	0.15 (.004)	0.70 (<.001)
**15**	<0.01 (.955)	0.65 (.002)
**16**	0.02 (.795)	0.59 (.007)
**17**	0.11 (.047)	0.76 (<.001)
**18**	-0.03 (.586)	0.71 (<.001)
**19**	0.03 (.580)	0.70 (<.001)
**20**	0.05 (.356)	0.65 (.002)
**21**	-0.11 (.033)	0.52 (.030)
**22**	0.12 (.022)	0.67 (.001)
**23**	0.07 (.201)	0.68 (<.001)
**Judgment**	0.41 (<.001)	0.48 (.065)
**Overall score**	0.19 (<.001)	0.88 (<.001)

## Discussion

The widely adopted NG14 guidance^14^ on the management of melanoma is now considered partly outdated by expert consensus^15^. In light of advances in adjuvant treatment for stage III disease, experts have called for broader indications for sentinel lymph node biopsy (SLNB), and the findings of MSLT-2^9^ and DeCOG-SLT^8^ suggest that completion lymphadenectomy is not necessarily indicated in all patients with a positive SLNB. This guidance has been reflected in 14 out of 29^15^^,^^28^, ^29^, ^30^, ^31^, ^32^^,^^38^^,^^47^^,^^49^^,^^55^ guidelines published since NG14, although none of the guidelines reviewed in this study equaled NG14’s development methodology, as determined by the AGREE II instrument.

NG14^14^ outscored other guidelines because it included additional elements such as patient and public involvement in guideline creation, external review of recommendations, auditing criteria, and support for guideline implementation.

The AGREE II tool enables users to rank guidelines by methodological quality, but there are no empirical data to suggest guidelines with higher AGREE II scores achieve better clinical outcomes, and there is no guidance on what scores guidelines should achieve before their uptake in routine clinical practice. In the current study, authors had good concordance on determining which guidelines were of comparatively superior quality (Kendall's W statistic), but there was poor agreement on specific scores (Fleiss kappa statistic). This suggests that the AGREE II tool is reliable and appropriate for ranking guidelines against each other, though the precise scores vary considerably depending on the assessor and cannot be used to quantify differences in quality between guidelines.

Another limitation of the AGREE II tool is that it is largely limited to only assessing the methodological quality of guideline development and how well guidelines reflect current evidence is assessed only in one item. Guidelines can score highly even if they are outdated, as was the case with NG14^14^ and SIGN 146^50^ in this study. In addition, guidelines based on expert consensus can score poorly because they lack a systematic review of the evidence. This could lead to unfair exclusion of otherwise methodological rigorous consensus statements that have a valuable role to play in areas where evidence is scarce^26^^,^^27^^,^^43^.

## Conclusion

This paper suggests that guidelines published since NG14^14^ have not met the same methodological development standards. More updates to NG14^14^ are needed and these are planned for 2022^55^. A pragmatic approach should be taken to melanoma management, with careful consideration given to the results of landmark trials published since the development of NG14^14^.

## Declaration of Competing Interest

At the time of writing, Conrad J. Harrison was enrolled on the National Institute for Health and Care Excellence (NICE) scholarship program, and as such could receive expenses from NICE for attendance at NICE events. No specific funding was received for this work.

Chloe Jacklin, Matthew Tan and Sanskrithi Sravanam have no conflicts of interest to disclose.

## Appendix Supplementary materials

Image, application 1
