# Supplementary material for: Appraisal of International Guidelines for Cutaneous Melanoma Management using the AGREE II assessment tool
Source: JPRAS Open. 2021 Dec 8;31:114–22. doi: 10.1016/j.jpra.2021.11.002 (PMC8732330; doi:10.1016/j.jpra.2021.11.002)
Supplement: Supplementary file 1 [file mmc1.docx]

Supplementary 1. Search Strategies. Search strategy for (a) PubMed and (b) Medline.

(a) PubMed database was searched using the following search strategy (melanoma*[Title/Abstract]) AND (guideline*[Title/Abstract] OR guidance [Title/Abstract] OR recommendation*[Title/Abstract] OR consensus [Title/Abstract] OR initiative*[Title/Abstract])

August 2021

(b) Medline was searched using the following search strategy:

1. Melanoma/
2. (Melanoma* and manag*).ab,ti.
3. (Melanom* and treat*).ab,ti.
4. (melanoma* and intervention*).ab,ti.
5. 1 or 2 or 3 or 4
6. (guidance or guideline* or recommendation* or consensus).ti.
7. 5 and 6
8. Limit to 5 (guideline or practice guideline)
9. 7 or 8

March 2020
